# Idiopathic granulomatous hypophysitis: a systematic review of 82 cases in the literature

**DOI:** 10.1007/s11102-013-0510-4

**Published:** 2013-08-29

**Authors:** Benjamin H. M. Hunn, William G. Martin, Steven Simpson, Catriona A. Mclean

**Affiliations:** 1 School of Medicine, University of Tasmania, Hobart, TAS 7000 Australia; 2 Royal Hobart Hospital Tasmania, Hobart, TAS 7000 Australia; 3 Menzies Research Institute, University of Tasmania, Hobart, TAS 7000 Australia; 4 Department of Anatomical Pathology, The Alfred Hospital, Melbourne, VIC 3084 Australia

**Keywords:** Hypophysitis, Granulomatous, Lymphocytic, Systematic review

## Abstract

Idiopathic granulomatous hypophysitis (IGH) is a rare inflammatory disease of the pituitary. There is debate in the scientific literature as to whether IGH represents a continuum of disease with lymphocytic hypophysitis or has a distinct pathogenesis. Due to the rare nature of the disease, previous descriptions have been limited to single case reports or small series. In the present study, a systematic review of the literature was performed for cases of IGH. 82 cases met inclusion criteria. Data was gathered on IGH clinical aspects, in order to elucidate any associations useful in determining pathogenesis, appropriate clinical treatment, or prognosis. Univariate and multivariate analysis was performed on available data. Female sex was significantly associated with IGH (*p* < 0.0001). Fever (*p* = 0.002), nausea or vomiting at presentation (*p* = 0.031), and histological evidence of necrosis (*p* = 0.022) correlated with reduced time to presentation. Panhypopituitarism at presentation predicted need for long term hormone replacement (*p* = 0.014). Hyperprolactinaemia (*p* = 0.032), normal gonadal (*p* = 0.037) and thyroid axes (*p* = 0.001) were associated with reduced likelihood of long-term hormone replacement. Anorexia (*p* = 0.017), cold intolerance (*p* = 0.046), and fatigue (*p* = 0.0033) were associated with death from IGH. Patients who had excisional surgery alone trended towards increased rates of symptom resolution, compared with patients who received corticosteroids as an adjunct to excisional surgery (*p* = 0.11). This article details the first systematic review of IGH, and presents evidence for a female predilection of the disease. Implications for pathogenesis, and a suggested clinical approach are discussed. An online disease registry has been established to facilitate further IGH research.

## Introduction

**Fig. 1:**
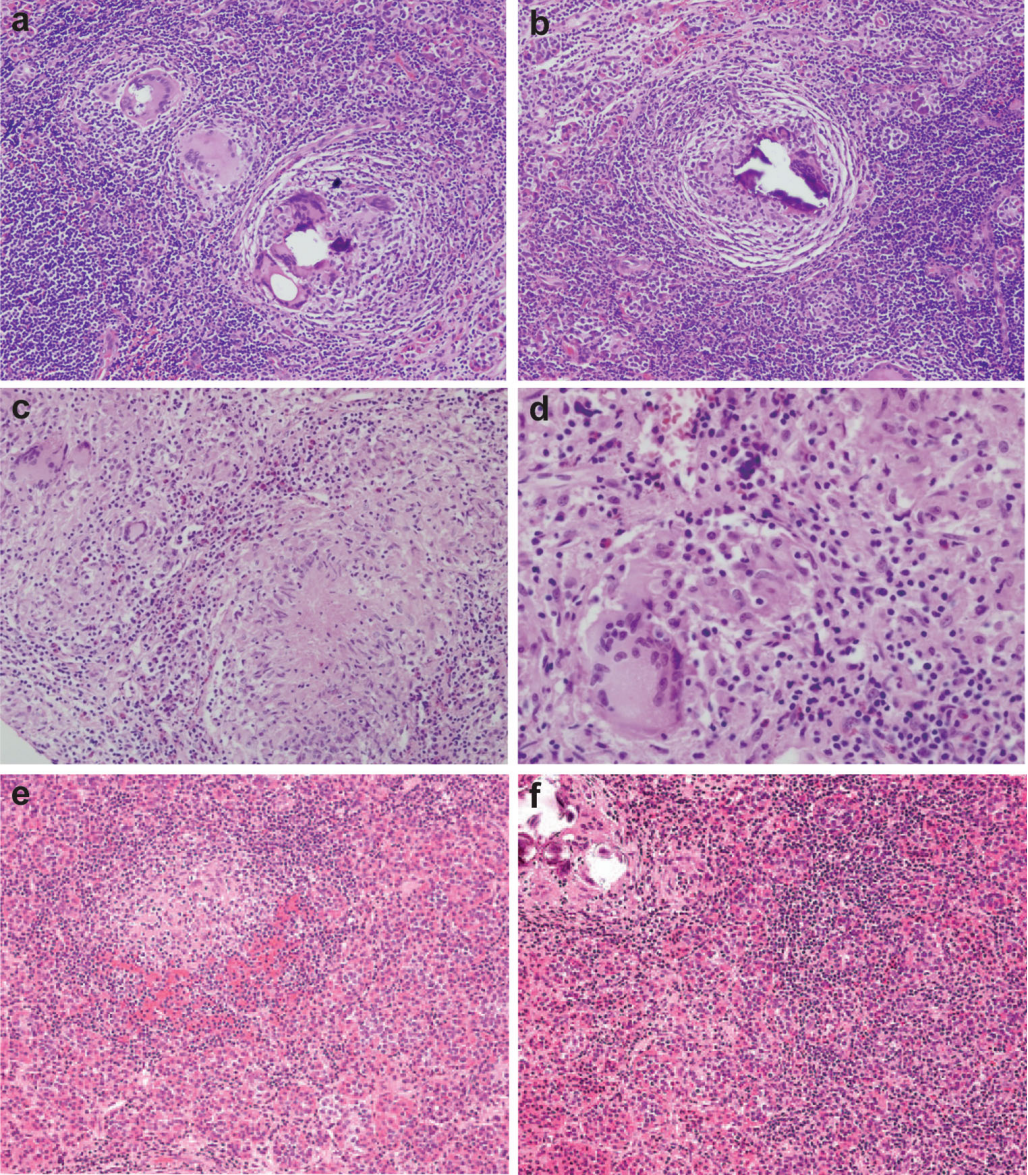
The histological spectrum of idiopathic granulomatous hypophysitis. Granulomatous hypophysitis is characterised by variable numbers of multinucleated giant cells and lymphoyctes, numerous histiocytes, some forming granulomas, and variable amounts of fibrosis. Figures **a** and **b** are taken from our own experience. Figures **c** and **d** reproduced from Lee et al. [28] under the Creative Commons license, and figures **e** and **f** are reproduced from Jastania et al. [24] with permission from Springer

Hypophysitis is a relatively rare disorder, with an estimated incidence of one case per 9 million people per year [5]. Histologically, hypophysitis is further divided into three pathological subtypes: lymphocytic, granulomatous, and xanthomatous hypophysitis [18, 41, 48]. Lymphocytic hypophysitis (LH) is the most commonly reported subtype, with the most recent comprehensive review of the literature listing 397 cases [7]. LH is characterised by an autoimmune lymphocytic infiltrate that can involve the anterior pituitary, the infundibulum and the posterior lobe of the pituitary (infundibulo-neurohypophysitis), or the entire pituitary gland [1, 7]. Granulomatous hypophysitis (GH) is likely to be the second most common subtype, and features “widely distributed multinucleated giant cells,” numerous stationary phagocytic cells (histiocytes), some forming granulomas, and variable amounts of lymphocytic infiltration and fibrosis (Fig. 1) [16, 49]. Xanthomatous hypophysitis was first described by Folkerth and her colleagues in 1998, and is characterised by infiltration of the anterior pituitary by “foamy histiocytes,” that contain lipid on electron microscopy [18].

Granulomatous hypophysitis can occur as a primary phenomenon, or secondary to systemic disease. Secondary causes of hypophysitis are numerous and varied, and include tuberculosis, sarcoidosis, syphilis, pituitary adenoma, Langerhan’s histiocytosis, Wegener’s granulomatosis, and Rathke’s cleft cyst rupture [4, 11, 21, 27, 30, 35, 37, 42]. The diagnosis of idiopathic granulomatous hypophysitis (IGH) is made following exclusion of secondary causes.

Idiopathic granulomatous hypophysitis was first described by Brissaud, Gougerot and Gy in 1908, and further defined by Simmonds in 1917 [3, 44]. Since the original descriptions there have been multiple individual cases reports and few case series published. Some authors have reviewed the subject of IGH, but none have done so in a systematic manner. The very rare nature of the disease effectively has thus far precluded prospective gathering of data, and limited analysis of patient populations to case reports and small series.

In this article, a systematic review of cases of IGH has been conducted, with a view to summarising the available literature. This informs a discussion of the differences and similarities of lymphocytic and IGH, and the relevance of these features to the underlying pathogenesis of the disease. An online disease registry has also been established to further facilitate systematic data collection of IGH cases.

## Materials and methods

### Literature searches

The online databases Medline (Pubmed), EMBASE, and Web of Science were searched on the 10th of February, 2013, for all documents containing the words “granulomatous hypophysitis,” which returned 74, 148, and 131 results respectively. Articles were included from all time periods. A manual search of the literature was also performed. Articles were excluded if (1) they were reviews with no new cases, (2) granulomatous hypophysitis was not histologically confirmed, (3) they were cases series without individualised data, (4) cases were reported in languages other than English or (5) they reported granulomatous hypophysitis with a secondary etiology. Cases reported as “granulomatous and lymphocytic hypophysitis” or similar were included. Cases that were repeated in two different articles were included only once. One case that was reported as idiopathic GH but is considered by the authors of this review to represent likely tuberculous GH was excluded [2]. A list of all reviewed articles is available as supplementary data at www.granulomatoushypophysitis.org.

### Classification of cases and data collection

Idiopathic, or primary, cases of granulomatous hypophysitis were classified based on the absence of evidence of a systemic disease or infection, as reported by the relevant authors. Clinical symptoms were classified into groups. For example, menstrual changes included individuals reported as suffering from oligomenorrhoea and amenorrhoea. The duration of symptoms was rounded to the nearest month. Biochemical abnormalities were summarised as high, low, or normal, where these data were available. Where only raw data was available, these were classified as high, low, or normal based on the reference ranges available in the Royal Australasian College of Pathologists test manual (available at www.rcpamanual.edu.au). Cases were classified as exhibiting panhypopituitarism if described as such by the reporting authors, or if deficient in 3 or more anterior pituitary axes. One case reporting a patient who was 11 days post-partum with serum prolactin in the normal range was classified as hypoprolactinaemic, based on prolactin ranges described by Riordan and Wambach [15, 40].

### Data analysis

Significance of variables from expected distributions (evenly divided between categories) were assessed by binomial regression for dichotomous variables and Chi squared goodness of fit test for multilevel variables. Significance in difference of continuous variables by sex was assessed by linear regression. Where necessary, dependent continuous variables were transformed to reduce heteroskedasticity, but all coefficients are reported on the normal scale. Significance of difference in occurrence of outcome (death, recurrence, remission) was assessed by logistic regression. Data analysis was performed using STATA 12.1 (StataCorp, Texas, USA) and GraphPad Prism 6 (Graphpad Software, California, USA).

## Results

### Demographics

**Fig. 2:**
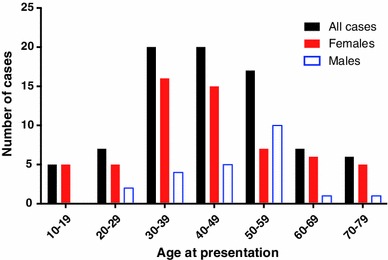
Age at presentation of idiopathic granulomatous hypophysitis

82 cases were analysed. 59 (72.0 %) were female, demonstrating a statistically significant female predilection of IGH, based on the available data (*p* < 0.0001). A statistically significant gender difference persisted when case series without individual data were included in the analysis, and also when data was compared against a 51:49 female:male ratio (data not shown). Age data are shown in Fig. 2. The mean age at presentation was 44.4 (SD 15.0) years. The mean age of females at presentation was 43.0 (SD 15.9) years, and of males was 48.0 (SD 12.0) years. There was no statistically significant difference in the age of presentation between genders (*p* = 0.17).

### Clinical features

Data was available on symptom duration for 63 patients (76.8 %). The median duration of symptoms was 8 months (interquartile range 2–24), this not differing by sex (*p* = 0.26). Presenting features are summarised in Table 1. The most common presenting symptom was headache, reported in 61.0 % (n = 50/82) of cases, followed by visual changes, reported in 41.5 % (n = 34/82) of cases. Fever at presentation was significantly predictive of a shorter duration of illness prior to presentation [average 6.09 months shorter (95 % CI −9.89, −2.29) *p* = 0.002], as was nausea or vomiting at presentation [average 5.01 months shorter (95 % CI −9.55, −0.46) *p* = 0.031], while headache showed a negative trend [average 6.92 months shorter (95 % CI −14.95, 1.12) *p* = 0.092]. Impotence among males was associated with a longer duration of symptoms until presentation, on average 27.14 months longer [(95 % CI 9.04, 45.24), *p* = 0.003].

**Table 1:** Presenting features of primary granulomatous hypophysitis

Presenting symptom or sign	n/N (%)
Headache	50/82 (61.0)
Visual changes	33/82 (40.2)
Polyuria or polydipsia	22/82 (26.8)
Cranial nerve palsies^a^	22/82 (26.8)
Cranial nerve II compression	12/82 (14.6)
Cranial nerve III	12/82 (9.8)
Cranial nerve IV	1/82 (1.2)
Cranial nerve VI	6/82 (7.3)
Cranial nerve VII	2/82 (2.4)
Fatigue	21/82 (25.6)
Nausea or vomiting	18/82 (22.0)
Anorexia	11/82 (13.4)
Fever	8/82 (9.8)
Cold intolerance	7/82 (8.5)
Galactorrhoea	6/82 (7.3)
Symptoms analysed only for one sex	
Menstrual changes (female)	26/59 (45.6)
Impotence or decreased libido (male)	7/23 (30.4)

^a^Some cases reported multiple cranial nerve palsies

Information regarding associated diseases was also gathered. 5 cases (6.1 %) were reported to be associated with autoimmune diseases: ulcerative colitis and thyroiditis [16], thyroiditis [25], pyoderma gangrenosum [8], Crohn’s disease [14], and psoriasis [9]. Females cases were examined for information regarding parity, which was available in 33.9 % (n = 20/59) of female patients. In four of these cases, it could only be discerned that gravidity and parity were at least equal to one or two [10, 15, 23, 33]. In those cases where full data on parity where available (n = 20/59), the mean parity was 1.2 (SD 0.89).

### Biochemical features

Biochemical data was available for 70.7 % (n = 58/82) of cases, and is summarised in Table 2. The most common biochemical abnormality was supressed hypothalamic–pituitary–adrenal axis (low ACTH or cortisol), described in 73.1 % of cases where data was given (n = 38/52). Supressed growth hormone (low GH or IGF-1), was reported in 66.7 % of cases where given (n = 20/30) and a suppressed gonadal axis (low FSH or LH) present in 66.0 % of cases where reported (n = 35/53). Testing for anti-pituitary auto-antibodies was performed in 5 cases (6.1 %) and was positive in 2 cases (2.4 %).

**Table 2:** Biochemical features of granulomatous hypophysitis

Endocrine axis	Number of reports assessing axis (%)	Number of reports where data was available (%)		
		Axis supressed	Axis normal	Axis elevated
Growth hormone (GH or IGF-1)	30/82 (36.6)	20/30 (66.7)	9/30 (30.0)	1/30 (3.3)
HPA (ACTH or cortisol)	52/82 (63.4)	38/52 (73.1)	14/52 (26.9)	0 (0)
Gonadal (FSH or LH)				
Males	14/23 (60.9)	10/14 (71.4)	4/14 (28.6)	0 (0)
Females	39/59 (66.1)	25/39 (64.1)	14/39 (35.9)	0 (0)
Combined	53/82 (64.6)	35/53 (66.0)	18/53 (34.0)	0 (0)
Thyroid (TSH or T4)	54/82 (65.9)	35/54 (64.8)	19/54 (35.2)	0 (0)
Prolactin	54/82 (65.9)	7/54 (13.0)	19/54 (35.2)	28/54 (51.9)
Other endocrine abnormalities		Number of cases (%)		
Laboratory diagnosis of diabetes insipidus reported		22/82 (26.8)		
Panhypopituatirism		40/82 (48.8)		

*GH* growth hormone, *IGF*-*1* insulin-like growth factor 1, *HPA* hypothalamic–pituitary–adrenal, *ACTH* adrenocorticotropic hormone, *FSH* follicle stimulating hormone, *LH* lutenising hormone, *TSH* thyroid stimulating hormone, *T4* thyroxine

### Radiological features

Radiological data were available for 90.2 % of cases (n = 74/82). Pituitary enlargement was the most common feature, reported in 93.2 % (n = 69/74) of cases. 66.2 % (n = 49/74) described suprasellar extension of the pituitary gland, including 25.7 % (n = 19/74) that demonstrated extension to, or compression of, the optic chiasm. A thickened infundibulum was present in 33.8 % of cases (n = 25/74). Pituitary contrast enhancement on computed tomography was reported in 24.3 % of cases (n = 18/74), and cystic change within the pituitary gland was present in 4.0 % (n = 3/74) of cases. Magnetic resonance imaging (MRI) was reported in 62.2 % of cases (n = 51/82). MRI-specific data was poorly reported. Of those patients who had an MRI, 29.4 % (n = 15/51) were reported to have T1-weighted isointensity of the pituitary gland and 3.9 % (n = 2/51) reported T1 hypointensity. Ten cases (19.6 %) reported loss of the “posterior pituitary brightspot” on T1-weighted MRI. T2-weighted imaging was reported as hyperintense in 8 cases (15.7 %), isointense in 4 cases (7.8 %), and hypointense in 1 case (2.0 %).

### Histological features

All cases had some histological data reported. 76 cases reported histopathology from surgical specimens and 6 cases were from autopsy. 62.2 % (n = 51/82) reported lymphocytic infiltration of the pituitary gland, and 61.0 % (n = 50/82) described macrophagic infiltration. Plasma cells were present in 31.7 % (n = 26/82) of cases, fibrosis in 19.5 % (n = 16/82), and necrosis in 14.6 % (n = 12/82). Patients who had necrosis on histology had a significantly shorter duration of symptoms prior to presentation [average 5.90 months shorter (95 % CI −10.96, −0.85) *p* = 0.022]. 60.0 % (n = 49/82) of cases reported the presence of giant cells. Of these, 73.5 % (n = 36/49) reported multinucleate giant cells of unspecified type, and 26.5 % (n = 13/49) reported Langhans type giant cells.

### Tuberculosis exclusion

70 cases (85.3 %) described one or more methods of excluding *Mycobacterium tuberculosis* (TB) as the aetiological agent in GH. Most commonly this was a pituitary tissue Ziehl-Neelsen (ZN) stain (81.4 %, n = 57/70), which was negative in all reports where it was given. A Mantoux test was performed as the sole TB test in 2 cases, which in one case was positive [43]. 15 cases (21.4 %) reported both a ZN stain and a Mantoux test. 11 cases (13.4 %) used other methods of excluding TB infection. 5 cases (6.1 %) utilised molecular methods of excluding TB infection, either tissue or cerebrospinal (CSF) TB polymerase chain reaction (PCR), or interferon-gamma release assays (e.g. QuantiFERON^®^-TB Gold). In 6 cases (7.3 %), the authors stated that they excluded systemic infection as the underlying aetiology of GH but did not explain the methods used. A subgroup analysis was performed on the 5 cases of IGH where TB infection had been excluded using molecular methods. This group contained 2 males and 3 females, with a mean age of 43.8 (SD 9.6) years. There were no significant differences between the unequivocal cases and the remainder of the data-set.

### Treatment

Data on treatment modalities were available in 64/82 cases (78.1 %). The majority of cases were treated with excisional pituitary surgery alone (46.9 %, n = 30/64), excisional surgery and corticosteroids (35.9 %, n = 23/64), or pituitary biopsy and corticosteroids (14.1 %, n = 9/64). 2 patients (3.1 %) had pituitary biopsy alone, and further treatment was not discussed. A comparison of treatment modalities and their associated clinical outcomes in this sample is provided in Table 3.

**Table 3:** Comparison of outcomes between cases managed with excision and excision and corticosteroids, and excision and biopsy and corticosteroids

Outcome	n/N (%)		
	Excision only (n = 30/82)	Excision and corticosteroids (n = 23/82)	*p* value
Symptom resolution	19/21 (90.5)	13/19 (68.4)	*p* = *0.098*
Need for hormone replacement therapy	17/26 (65.4)	18/20 (90.0)	*p* = *0.067*
Recurrence	2/25 (8.0)	3/20 (15.0)	*p* = *0.46*

Outcome	n/N (%)		
	Excision only (n = 30/82)	Biopsy and corticosteroids (n = 9/82)	*p* value
Symptom resolution	19/21 (90.5)	7/8 (87.5)	*p* = *0.82*
Need for hormone replacement therapy	17/26 (65.4)	8/9 (88.9)	*p* = *0.21*
Recurrence	2/25 (8.0)	1/9 (11.1)	*p* = *0.78*

Significance of difference in outcome by treatment group assessed by logistic regression

### Outcomes

Duration of follow up was reported for 43/82 cases (52.4 %). The median duration of follow up was 18.0 months (interquartile range 8–36), this not differing by sex (*p* = 0.51). 53.7 % (n = 44/82) of all cases required long-term hormone replacement for one or more pituitary hormones. Panhypopituitarism at presentation predicted need for long term hormone replacement [OR 6.44 (95 % CI 1.46, 28.53), *p* = 0.014]. Galactorrhoea [OR 0.07 (95 % CI 0.01, 0.75), *p* = 0.028], hyperprolactinaemia [OR 0.09 (95 % CI 0.01, 0.81), *p* = 0.032], normal gonadal axis [OR 0.18 (95 % CI 0.04, 0.91), *p* = 0.037], and euthyroidism [OR 0.06 (95 % CI 0.01, 0.34), *p* = 0.001] at presentation were all associated with reduced requirement for long-term hormone replacement. Both recurrence and death from disease were relatively infrequent in this cohort (n = 6 for both). Death was significantly predicted by anorexia or weight loss at presentation [OR 8.50 (95 % CI 1.46, 49.41), *p* = 0.017], cold intolerance at presentation [OR 7.10 (95 % CI 1.04, 48.65), *p* = 0.046], and fatigue at presentation [OR 6.94 (95 % CI 1.17, 41.20), *p* = 0.033]. These associations did not persist when outcome data was corrected for year of publication, as all deaths occurred prior to 1969. There was a longer duration between symptom onset and diagnosis among those who died, on average 18.2 months longer, though this difference did not reach significance (*p* = 0.18). No clinical or demographic parameters significantly predicted recurrence of disease.

## Discussion

This systematic review presents the largest number of IGH cases ever enumerated, with 82 cases meeting the inclusion criteria. Prior to this report, up to six cases had been reported at any one time, in two different case series [19, 38]. One of the key concerns of researchers and treating physicians is differentiation of IGH from lymphocytic hypophysitis. Caterugli and colleagues reviewed 379 cases of LH, finding features of LH to be quite disparate to those of IGH cases demonstrated in our sample. Headache was present in fewer LH patients compared with IGH patients (45.9 vs. 61.0 %; *p* < 0.05), and more LH patients were hypoprolactinaemic (19.5 vs. 50.9 %; *p* < 0.05). Radiology and treatment data provided by the review by Caterugli and colleagues were descriptive in nature and thus could not be compared to our data.

There are differing perspectives on the pathogenesis of idiopathic GH. One of the key controversies centres on whether LH and IGH represent separate diseases, or are temporally distinct observations of the same disease. In 1984, McKeel observed that “…there may be a spectrum of disease, with purely lymphocytic adenohypophysitis being the predominant early lesion, and the granulomatous component being the later component of the same disease process” [31]. In contrast, Caterugli and colleagues suggest that GH “…appears distinct from lymphocytic hypophysitis because it lacks key epidemiological features that are present in lymphocytic hypophysitis, such as female bias, association with pregnancy, occasional spontaneous resolution, and association with other well-established autoimmune diseases” [7]. Our data demonstrates a female predilection of IGH, and shows that most cases of IGH exhibit lymphocytic infiltration of the pituitary gland. In addition, the mean age of presentation of females in this data series is 43 years, some 8 years subsequent to the mean age of presentation in LH [7]. These findings support the suggestion that IGH may represent a later manifestation of inflammatory hypophyseal disease than LH, perhaps in a subset of patients that first developed subclinical LH. Indeed, in a mouse model of lymphocytic hypophysitis, some test animals developed multinucleated giant cells similar to those seen in granulomatous hypophysitis [51].

The pathological mechanisms underpinning autoimmune pituitary disorders merit consideration. There is evidence in the published literature that anti-pituitary auto-antibodies (APA) are most likely to play a role in the pathogenesis of autoimmune pituitary disorders. Previous reports have demonstrated elevated levels of APA in lymphocytic hypophysitis, but also in empty sella syndrome, isolated pituitary hormone deficiencies, panhypopituitarism, and individuals with isolated hyperprolactinaemia [20, 26, 36, 45–47]. However, it should be noted that elevated serum APA have also been reported in conditions as diverse as eating disorders and cryptorchidism [17, 39]. The antigenic targets of APA have been variously identified as alpha-enolase, growth hormone, pituitary gland specific factors 1a and 2, secretogranin II, and chorionic somatomammotropin [12, 32, 36, 45–47]. In our study, 5 cases included examination for evidence of APA, and in 2 of these APA were demonstrated. These papers that published data on APA were early studies, using earlier immunological methods that are poorly sensitive and specific. In addition, assays investigating for APA may not target all autoantibodies, and therefore a finding of absent APA does not necessarily indicate absence of autoantibodies. It is possible that there is more than one autoimmune pituitary target, and that these different targets may cause different, but overlapping, forms of pituitary inflammation.

The majority of cases of IGH become apparent due to mass effects from an enlarged pituitary gland, prompting the need for surgical resection of the gland. It follows, therefore, that a large number of IGH cases may be subclinical, or manifest in ways that are not attributable to mass effects. The two most clinically significant manifestations are central diabetes insipidus and hypopituitarism. In approximately one-third of central diabetes insipidus cases the cause remains unknown, and treatment consists of vasopressin replacement when deficiency becomes clinically apparent [6, 34]. There is a vein of literature linking lymphocytic infundibuloneurohypophysitis to the pathogenesis of central diabetes insipidus [22, 34]. In addition, many authors have detailed an association between infundibular swelling on imaging and the development of central diabetes insipidus [13, 29, 34, 50]. Both central diabetes insipidus and infundibular enlargement were reported in approximately one-third of IGH cases in this review, indicating that IGH may account for a portion of central diabetes insipidus cases that are at present considered idiopathic. Similarly, IGH may account for some cases of panhypopituitarism that remain cryptogenic despite intensive investigation. Further autopsy series may help to better understand the presence of undetected pituitary disorders underlying cases of central diabetes insipidus and panhypopituitarism.

Despite diagnostic advances, separating primary and secondary cases of GH remains fraught with difficulty. In cases where GH is suspected as a diagnosis, all available steps to exclude infection or systemic disease as the aetiology of the condition should be taken. This would include, where resources allow, Mantoux testing, chest radiography, TB PCR of cerebrospinal fluid and pituitary tissue, serum syphilis testing and measurement of serum angiotensin converting enzyme. Many of the patients described as suffering from IGH did not have all or even some of these investigations reported, and this may indicate that our data set includes some patients that were suffering from secondary GH. In settings where TB is endemic and resources are limited, empirical treatment for TB may represent a prudent course of action, even if no causative organisms can be demonstrated on histopathology. Our data trended towards increased rates of symptom resolution when patients were treated with surgical excision, compared with surgical excision combined with corticosteroid treatment. These findings may represent an advantage of surgical excision alone as a treatment, or may demonstrate that corticosteroids are added to surgical excision only in the most severe IGH cases. There were no significant differences between patients who had surgical excision compared with those who had a pituitary biopsy and corticosteroid treatment. With these data in mind, it may be appropriate to proceed with pituitary biopsy in the first instance, with excisional surgery as an option in those patients who fail to respond.

There are a number of limitations to this study. Reporting bias favouring a specific patient group, for example females, may give an inaccurate impression of IGH demographics. Similarly, case reports of more severe or unusual cases may be more likely to be written and published, providing data that indicates a more severe disease phenotype than may be routinely seen in clinical practice. Cases where suspected hypophysitis was treated with corticosteroids but without histological confirmation were not included in this review, although this may represent an appropriate treatment strategy in suspected cases. Most cases where death was an outcome were from older reports, perhaps signifying that treatment, especially transphenoidal surgery, had not advanced sufficiently to successfully treat IGH. The relatively small number of cases in this review limits the power of this study to generate statistically significant evidence of disease associations. In part to help address some of these limitations, an online disease registry has been established at www.granulomatoushypophysitis.org—this registry operates under University of Tasmania ethics approval H0013267. Clinicians can register de-identified data of any IGH cases that will be stored on a secure server and accessible to researchers with an interest in IGH.

In conclusion, we describe here the largest accumulation of cases of IGH yet reported. In addition to providing a general picture of treatment and clinical outcomes over time, the distribution of cases by sex, and the evaluation of predictors of clinical outcomes, it also provides epidemiologic evidence that could help inform surveillance and treatment in the future. However, this method provides only a crude picture of the disease, a better one to be gleaned by implementation and utilisation of a central disease registry whereby clinical information can be recorded in a standardised fashion.
